# Molecular Detection of *Streptococcus pneumoniae* on Dried Blood Spots from Febrile Nigerian Children Compared to Culture

**DOI:** 10.1371/journal.pone.0152253

**Published:** 2016-03-23

**Authors:** Pui-Ying Iroh Tam, Nelmary Hernandez-Alvarado, Mark R. Schleiss, Fatimah Hassan-Hanga, Chuma Onuchukwu, Dominic Umoru, Stephen K. Obaro

**Affiliations:** 1 Division of Pediatric Infectious Diseases and Immunology, University of Minnesota Masonic Children’s Hospital, Minneapolis, Minnesota, United States of America; 2 Center for Infectious Diseases, Microbiology and Translational Research, Minneapolis, Minnesota, United States of America; 3 Department of Paediatrics, Aminu Kano Teaching Hospital, Kano, Nigeria; 4 Department of Paediatrics, Federal Medical Center, Keffi, Nasarawa, Nigeria; 5 Department of Paediatrics, Nyanya General Hospital, Abuja, Nigeria; 6 Department of Pediatrics, University of Nebraska Medical Center, Omaha, Nebraska, United States of America; Instituto Butantan, BRAZIL

## Abstract

**Background:**

Nigeria has one of the highest burdens of pneumococcal disease in the world, but accurate surveillance is lacking. Molecular detection of infectious pathogens in dried blood spots (DBS) is an ideal method for surveillance of infections in resource-limited settings because of its low cost, minimal blood volumes involved, and ease of storage at ambient temperature. Our study aim was to evaluate a *Streptococcus pneumoniae* real-time polymerase chain reaction (rt-PCR) assay on DBS from febrile Nigerian children on Whatman 903 and FTA filter papers, compared to the gold standard of culture.

**Methods:**

Between September 2011 to May 2015, blood was collected from children 5 years of age or under who presented to six hospital study sites throughout northern and central Nigeria with febrile illness, and inoculated into blood culture bottles or spotted onto Whatman 903 or FTA filter paper. Culture and rt-PCR were performed on all samples.

**Results:**

A total of 537 DBS specimens from 535 children were included in the study, of which 15 were culture-positive for *S*. *pneumoniae*. The rt-PCR assay detected *S*. *pneumoniae* in 12 DBS specimens (2.2%). One positive rt-PCR result was identified in a culture-negative specimen from a high-risk subject, and two positive rt-PCR results were negative on repeat testing. Six culture-confirmed cases of *S*. *pneumoniae* bacteremia were missed. Compared to culture, the overall sensitivities of Whatman 903 and FTA DBS for detection of *S*. *pneumoniae* were 57.1% (95% CI 18.4–90.1%) and 62.5% (95% CI 24.5–91.5%), respectively. Nonspecific amplification was noted in an additional 22 DBS (4.1%). Among these, six were positive for a non-*S*. *pneumoniae* pathogen on culture.

**Conclusions:**

Rt-PCR was able to detect *S*. *pneumoniae* from clinical DBS specimens, including from a culture-negative specimen. Our findings show promise of this approach as a surveillance diagnostic, but also raise important cautionary questions. Several DBS specimens were detected as *S*. *pneumoniae* by rt-PCR despite growth of a non-*S*. *pneumoniae* pathogen on culture. A precise definition of what constitutes a positive result is required to avoid falsely over-identifying specimens.

## Introduction

*Streptococcus pneumoniae*, a commonly isolated bacterial pathogen in otitis media, can also cause invasive disease such as bacteremia, bacteremic pneumonia and meningitis, and is associated with high case fatality rates. Nigeria, the most populous nation in Africa, is one of the countries with the highest pneumococcal mortality rates in the world; in 2000, there were an estimated 86,000 deaths in children under 5 years of age, the second highest of any country worldwide [1]. However, invasive pneumococcal disease (IPD) is likely being underreported, based on the high prevalence of pneumococcal carriage in Nigerian infants [2] and reports of substantial pneumococcal disease burden in other parts of sub-Saharan Africa [3–5].

A major challenge in accurately determining the prevalence of IPD is the lack of resources necessary for obtaining and processing diagnostic specimens. In addition, the widespread use of non-prescription antibiotics prior to evaluation by a physician makes recovery of viable bacteria difficult in children who present for care [6, 7]. Dried blood spot (DBS) testing is an ideal method for diagnosing infections in resource-limited settings because of its low cost, minimal blood volumes involved, and capacity for storage and transport at ambient temperature. Furthermore, molecular methods such as polymerase chain reaction (PCR) have been able to identify pneumococcus in culture-negative specimens obtained from subjects that have been pre-treated with antibiotic therapy [7, 8].

Whatman 903 filter paper has been the standard for DBS used in newborn screening for decades [9]. More recently, newer materials have been introduced in which the cellulose matrix has been impregnated with surfactant, a chelating agent, buffer, and a free radical trap [10]. These Flinders Technology Associates (FTA) filter papers have the ability to lyse cells on contact, denature proteins, and protect DNA from degradation. Whatman FTA has been shown to lead to lower cycle thresholds (i.e. a lower limit of detection (LLD) on real-time (rt)-PCR) in cerebrospinal fluid (CSF) studies compared to Whatman 903 [11]. However, studies comparing the performance of these filter papers for subsequent detection of *S*. *pneumoniae* genes are lacking.

The aim of our study was to evaluate a *S*. *pneumoniae* rt-PCR assay on DBS using different types of filter paper among a cohort of febrile Nigerian children. We hypothesized that DBS can be used to detect *S*. *pneumoniae* with high sensitivity and specificity, compared to the gold standard of culture. Our aim was to evaluate DBS on Whatman 903 and FTA from a clinical cohort of febrile Nigerian children using rt-PCR and determine whether this method can more accurately detect *S*. *pneumoniae* compared to the gold standard of culture.

## Materials and Methods

### Ethics statement

This study was approved by the Ethics Committees of the Federal Capital Territory, Federal Medical Center Keffi, and Aminu Kano Teaching Hospital, and by the University of Nebraska Medical Center and University of Minnesota Institutional Review Boards. Written signed informed consent was obtained from the parent or guardian.

### Bacterial strains

For the laboratory validation, clinical isolates of *S*. *pneumoniae* used were collected as part of the Active Bacterial Core surveillance of the Centers for Disease Control and Prevention’s (CDC) Emerging Infections Program [12]. These reference strains were collected from culture-confirmed cases of meningitis, pneumonia, or bacteremia, and cover 12 serotypes (1, 3, 4, 5, 6A, 7F, 9V, 14, 18C, 19A, 19F, 23F) present in the 13-valent pneumococcal conjugate vaccine. Nine bacterial species, including *Neisseria meningitidis*, *Haemophilus influenzae* type e, *H*. *influenzae* indeterminate type, *Streptococcus mitis*, methicillin-resistant *Staphylococcus aureus*, and Group A, B and G *Streptococcus*, were used as negative controls. All isolates were provided in saline suspensions of 0.5 McFarland [13].

### Samples and serial dilutions

The two papers selected for performance evaluation included the Whatman 903 and FTA classic cards (GE Healthcare Bio-Sciences, Pittsburgh, PA). *S*. *pneumoniae* isolates were suspended in phosphate buffered saline (PBS) to 0.5 McFarland, equivalent to 10^8^ CFU/mL [13]. These isolates were spiked into whole blood collected in sodium citrate tubes, and ten-fold serial dilutions of whole blood were prepared ranging from 0.5-5x10^4^ CFU/μL of blood. In addition, dilutions at 2 and 20 CFU/μL were prepared, based on previous studies identifying a detection limit in bacterial PCR within this range [14–16]. Hence, there were a total of 8 dilutions tested per serotype. Next, 100 μL from each dilution was spotted onto Whatman 903 and FTA filter paper until soaked through. Blood spots were allowed to dry overnight on the benchtop and then stored in sealed plastic bags at room temperature. We used as negative controls 9 strains of other bacterial species that are associated with invasive disease, as well as non-spiked samples of whole blood.

### DNA extraction

For whole blood, 200 μL of *S*. *pneumoniae* isolate was added to 100 μL of 0.04 g/mL lysozyme and 75 U/mL mutanolysin (Sigma-Aldrich Corp, St. Louis, MO) in Tris EDTA (TE, pH 8.5) for 1 hour at 37°C. Next, 20 μL of proteinase K (Qiagen, Valencia, CA) was added and the specimen was incubated at 56°C for 30 minutes. Following this step, 200 μL of QiaAmp Lysis (AL) lysis buffer (Qiagen) was added and the specimen was incubated for 10 minutes at room temperature. DNA was extracted using the QiaAmp DNA Mini Kit (Qiagen) according to the manufacturer’s instructions [17].

For each filter paper, three punches of DBS were collected, with each set of 3 mm punches on DBS followed by 15 punches on clean filter paper to prevent contamination between samples. For Whatman 903 DBS, three punches were placed into a 1.5 mL microcentrifuge tube and 200 μL of TE was added. The punches were washed by shaking at 40 rpm at 56°C over 16–20 hours. After washing, DBS were processed as previously described for whole blood. For DNA extraction from Whatman FTA DBS, three different methods including a TE buffer extraction method [18], a methanol extraction method [19], and a FTA purification reagent extraction method were assessed as described in S1 Appendix.

### Real-time PCR assay

Primers and probes previously developed by the CDC for detection of the *lytA* gene were used (S1 Table [17, 20]). Rt-PCR was performed using TaqMan Universal PCR master mix (Qiagen) and run on the LightCycler 480 (Roche Diagnostics, Indianapolis, IN). Each rt-PCR reaction consisted of a total volume of 23 μL, with 12.5 μL of the reconstituted master mix, and contained either 0.2 μM of primers and probe for *lytA*, or 0.32 μM primers and 0.08 μM probe for RNAse P. For rt-PCR assays of whole blood, 2 μL of the extracted DNA was used as template based on a standard protocol developed by the Minnesota Department of Health [21]. A template volume of 6.5 μL of extracted DNA was used for rt-PCR assays of DBS, in comparison to a template volume of 2 μL of extracted DNA from whole blood. This was done to correct for a smaller initial volume of blood used in DBS DNA extraction compared to whole blood. Rt-PCR reactions were cycled at 50°C for 2 minutes; 95°C for 10 minutes; and then 50 cycles at 95°C for 15 seconds and 60°C for 1 minute.

The LLD, defined as the lowest limit of detection in CFU/μL of blood, for each isolate was determined by the lowest dilution that was detectable on rt-PCR assay. DNA concentrations that yielded a cycle threshold (Ct) value of <40 on rt-PCR assay were defined as positive. Values ≥40 were considered equivocal and were repeated, and were considered positive if the repeat value was <40. The cutoff Ct value of 40 was chosen based on *in vitro* rt-PCR results on the spiked samples. For the laboratory validation, DBS samples were run in duplicate, and were counted as positive when both replicates tested positive. To demonstrate the presence and successful extraction of human cellular DNA and as a control for PCR inhibitors [22], rt-PCR assays were tested for the presence of the housekeeping gene RNase P on Whatman 903 and FTA DBS for a limited number of serotypes.

### Clinical study sites and participants

Children 5 years of age or under who presented to six hospital study sites throughout northern and central Nigeria were prospectively screened from September 2011 through May 2015, and enrolled if they had a fever >38.5°C associated with difficulty breathing or altered consciousness. In addition, a subgroup of healthy neonatal controls were also enrolled. A detailed questionnaire was administered by a physician to obtain information on clinical history and physical examination findings. After informed consent was obtained, the skin was cleaned with alcohol swabs and 1–3 mL of blood was directly collected and inoculated into an aerobic Bactec (Becton Dickinson, Temse, Belgium) culture bottle and delivered to the regional microbiology laboratory within 4 hours of collection for processing. In addition, the finger was cleaned with an alcohol swab before being pricked, and blood was spotted onto either five spots (diameter 1.3 cm) on Whatman 903, or one spot (diameter 2.5 cm) on Whatman FTA. Whatman 903 was available for the majority of the study period at the study sites, however in the later period these were replaced by Whatman FTA. DBS were stored in a -80°C freezer until they were transported to the University of Minnesota for further processing.

### Clinical laboratory methods

Aerobic blood culture bottles were held in the Bactec 9050 incubator (Becton Dickinson) for a maximum of 5 days. Positive cultures were subcultured using sheep blood, chocolate or McConkey agar. Subcultured plates were incubated under aerobic and 5% CO_2_ conditions at 35°C for 18–24 hours. *S*. *pneumoniae* isolates were identified by susceptibility to optochin and bile solubility [23].

For *S*. *pneumoniae* DNA amplification, each clinical specimen was run in triplicate for detection of *lytA* gene. Based on our validation studies, we defined a positive result for *S*. *pneumoniae* as a specimen that yielded a Ct value of <40 in 2/3 replicates by rt-PCR. Values ≥40 were considered equivocal and were repeated, and were considered positive if the repeat value was <40 in 2/3 replicates. Each specimen was tested for the presence of the housekeeping gene RNase P to demonstrate the presence and successful extraction of human cellular DNA [22].

### Data analysis

All data management and analysis was performed using Microsoft Excel 2011 (v.14.4.5, Redmond, WA). Exact binomial distribution was used to calculate 95% confidence intervals. Chi-square test was performed, with statistical significance set at 5%.

## Results

### High detection rate of pneumococcal DNA in spiked samples on both filter papers by rt-PCR assay

Amplification curves on rt-PCR for a representative dilution series demonstrated the integrity of the assay (S1 Fig). We detected pneumococcal DNA in 91/96 spiked samples (94.8% [95% CI 88.4–97.8%]). Five dilutions were not detected in whole blood by rt-PCR. Compared with spiked whole blood, pneumococcal DNA was detected in 83.5% (95% CI 74.6–89.8%) of Whatman FTA DBS, compared to detection in 81.3% (95% CI 72.1–88%) of 903 DBS. All of the non-pneumococcal samples were negative for *S*. *pneumoniae* DNA.

### Comparable range of LLD for Whatman 903 DBS compared with FTA DBS

The LLD on Whatman 903 DBS ranged from 0.5 CFU/μL of blood for 1/12 serotypes to 20 CFU/μL for 3/12 serotypes (Table 1). On Whatman FTA DBS the LLD ranged from 0.5 CFU/μL for 2/12 serotypes to 20 CFU/μL for 1/12 serotypes. On spiked whole blood, the LLD ranged from 0.5 CFU/μL for 8/12 serotypes to 5 CFU/μL for 1/12 serotypes.

**Table 1:** Comparison of LLD on whole blood versus Whatman 903 and FTA DBS.

Serotype	Whole blood		Whatman 903 DBS		Whatman FTA DBS	
	LLD (CFU/μL of blood)	Mean Ct value	LLD (CFU/μL of blood)	Mean Ct value	LLD (CFU/μL of blood)	Mean Ct value
1	0.5	38.79	5	38.08	5	37.04
3	5	38.68	5	38.35	5	39.8
4	0.5	37.81	5	39.1	5	39.85
5	0.5	37.53	2	37.57	2	39.3
6A	0.5	38.57	20	37.33	5	39.01
7F	2	38.35	5	39.13	5	38.94
9V	0.5	36.57	0.5	38.93	0.5	38.06
14	0.5	34.84	5	36.96	5	38.14
18C	0.5	38.77	5	37.07	2	39.61
19A	0.5	35.82	0.5	38.21	2	40.37
19F	2	38.52	20	37.77	5	39.1
23F	2	37.14	20	37.89	20	38.59

CFU, colony forming unit; Ct, cycle threshold; DBS, dried blood spots; LLD, lower limit of detection.

### Demographics of clinical DBS specimens

A total of 537 DBS specimens from 535 children were included in the study. Complete data was available for 261 participants (73.1%; Table 2). Of these, the median age was 14 months with the largest cohort being those from 12–59 months (49.0%), and 53.3% were males. The most common clinical diagnoses on admission were malaria (31.8%) followed by sepsis (29.9%) and respiratory tract illness/pneumonia (24.1%).

**Table 2:** Characteristics of study participants.

Characteristic	N = 261 (%)
Median age, months (range)	14 (0–132)
<2 months	57 (21.8)
2-<12 months	54 (20.7)
12-<60 months	128 (49.0)
60 months+	22 (8.4)
Male sex	139 (53.3)
Blood culture	
No growth	169 (64.8)
Contaminants*	44 (16.9)
*Salmonella* Typhi	15 (5.7)
*S*. *pneumoniae*	8 (3.1)
*Salmonella* spp.	7 (2.7)
*S*. *aureus*	3 (1.1)
*Escherichia coli*	3 (1.1)
*Candida* spp.	2 (0.8)
*Chryseomonas luteola*	2 (0.8)
Miscellaneous**	8 (3.1)
Diagnosis at admission	
Malaria	83 (31.8)
Sepsis	78 (29.9)
Respiratory tract illness/pneumonia	63 (24.1)
Diarrhea/dysentery/enteritis	10 (3.8)
Tonsillitis/pharyngitis	7 (2.7)
Enteric fever	5 (1.9)
Skin infection	4 (1.5)
Meningitis	3 (1.1)
Cardiac disease	3 (1.1)
Miscellaneous***	5 (1.9)

*Contaminants: alpha-hemolytic *Streptococcus*, *Bacillus* spp., coagulase-negative *Staphylococcus*, *Micrococcus* spp., non-hemolytic *Streptococcus*, *Pantoea*

**Citrobacter spp., *Enterobacter* spp., *Enterococcus* spp., *Haemophilus* spp., *Klebsiella* spp., *Pseudomonas* spp., *Salmonella* Paratyphi

***Dehydration, Neonatal tetanus, Osteomyelitis, Sickle cell crisis, Viral.

### Molecular detection of *S*. *pneumoniae* in clinical DBS compared to culture

Among the 537 clinical DBS specimens, including 481 Whatman 903 and 56 FTA, rt-PCR assay detected *S*. *pneumoniae* in 12 DBS specimens (2.2%). Five DBS were on Whatman 903 and seven were FTA. Nonspecific amplification was noted in an additional 22 DBS (4.1%).

Culture data were available for 436 isolates (81.2%; S2 Table). The majority of blood cultures had no growth (61.6%), and 15 were culture-positive for *S*. *pneumoniae* (3.4%). Among these culture-positive samples, *S*. *pneumoniae* was detected by rt-PCR in 9 DBS (60%), and nonspecific amplification was noted in one additional DBS that did not meet criteria for a positive result. Only two patients had blood collected on both Whatman 903 and FTA, and in each case both were culture-positive for *S*. *pneumoniae* and both types of DBS were positive by rt-PCR.

*S*. *pneumoniae* was detected by rt-PCR in an additional three specimens. One was a Whatman FTA DBS from a high-risk subject whose blood culture had no growth. On repeat rt-PCR testing the specimen again had Ct values <40 in 3/3 replicates. However, the other two positives were from a healthy neonatal control, and from a specimen that was culture-positive for *H*. *influenzae* type b. Both of these latter two specimens on repeat rt-PCR testing were negative.

For the other 22 DBS (64.7%) where *S*. *pneumoniae* was detected by rt-PCR but did not meet criteria for a positive result, two DBS detected *S*. *pneumoniae* in 2/3 replicates, but both of those Ct values were not <40. Furthermore, both of these clinical DBS were positive by culture for other bacteria (*S*. *aureus* and coagulase-negative *Staphylococcus*). *S*. *pneumoniae* was detected in 1/3 replicates of 20 DBS by rt-PCR, but only 11 had Ct values <40. Of these 11, one had no culture data, 8 had no growth on culture, and two were positive for *Salmonella* Typhi on culture.

The overall sensitivity of DBS was 60% (95% CI 32.3–83.7%) and specificity was 99.4% (95% CI 98.3–99.9%; Table 3). Both Whatman 903 and FTA showed similar performance for detection of *S*. *pneumoniae* from clinical DBS, with a sensitivity of 57.1% (95% CI 18.4–90.1) and specificity of 99.8% (95% CI 98.8–100) for Whatman 903, and sensitivity of 62.5% (95% CI 24.5–91.5) and specificity of 95.8% (95% CI 85.7–99.5%) for Whatman FTA.

**Table 3:** Sensitivity and specificity for blood culture and real-time PCR assays with Ct values.

		*S*. *pneumoniae* culture-positive			*S*. *pneumoniae* culture-negative		
		Total	Whatman 903,^a^ no. (mean Ct±SD)	Whatman FTA,^a^ no. (mean Ct±SD)	Total	Whatman 903, no. (mean Ct±SD)	Whatman FTA, no. (mean Ct±SD)
	*S*. *pneumoniae* PCR-positive^b^	9	4 (35.63±0.45)	5 (35.89±0.50)	3	1^c^ (39.1±0.73)	2^d^ (39.28±0.66)
	*S*. *pneumoniae* PCR-negative	6	3	3	521	473	46
Culture as gold standard	Sensitivity, % (95% CI)	60 (32.3–83.7)	57.1 (18.4–90.1)	62.5 (24.5–91.5)	-	-	-
Culture as gold standard	Specificity, % (95% CI)	-	-	-	99.4 (98.3–99.9)	99.8 (98.8–100)	95.8 (85.7–99.5)

Ct, cycle threshold; DBS, dried blood spot; rt-PCR, real-time polymerase chain reaction; SD, standard deviation

^a^Either Whatman 903 or FTA was available at the field site; subjects did not have DBS collected on both.

^b^*S*. *pneumoniae* PCR-positive detection was defined as *lytA* gene detected by rt-PCR with Ct value <40 in 2/3 replicates.

^c^Clinical DBS specimen was positive for *H*. *influenzae* type b on culture. The specimen was negative on repeat rt-PCR testing.

^d^One subject was a high-risk febrile patient, the second subject was a healthy neonatal control. On repeat rt-PCR testing, the first specimen was positive (Ct <40 in 3/3 replicates), the second specimen was negative.

## Discussion

This study provides the first description of molecular methods on DBS in a clinical cohort of febrile Nigerian children to detect bacterial pathogens. As all specimens were paired and tested by culture and rt-PCR, we were able to compare their performance. We were able to detect *S*. *pneumoniae* in DBS by rt-PCR, with an overall sensitivity of 60% (95% CI 32.3–83.7%) and specificity of 99.4% (95% CI 98.3–99.9%) compared to culture. The disappointing sensitivity of both filter papers (57.1% for Whatman 903 and 62.5% for FTA) for the detection of pneumococcal DNA was most likely due to the small amounts of whole blood captured in filter paper. Nonetheless, rt-PCR led to the identification of an additional *S*. *pneumoniae* specimen from a Whatman FTA DBS in a high-risk subject.

Our results, in particular the identification of *S*. *pneumoniae* in a culture-negative specimen, support the promise of molecular diagnostics. Molecular methods, including nucleic acid amplification tests, have the ability to detect minute amounts of nucleic acid of a pathogen, and studies using PCR to detect pneumococcal meningitis have shown sensitivity and specificity rates of 92–100% and 100% compared to the gold standard of culture, respectively [24, 25]. The method also does not require a viable microbe, and hence is less likely to be affected by prior antimicrobial therapy than culture-based methods. This was illustrated in Brazil, where rt-PCR was incorporated into routine public health surveillance of bacterial meningitis. Researchers found that using this method increased the detection of bacteria by up to 85% [8]. In bacteremic pneumonia, a study evaluating DBS PCR identified a three-fold increase in pneumococcal-positive specimens [26].

Nonspecific amplification was noted in almost 5% of clinical DBS, including DBS that were culture-positive for other bacteria, such as *S*. *aureus*, coagulase-negative *Staphylococcus*, alpha-hemolytic *Streptococcus* and *Salmonella* Typhi. When we compared Ct values of these DBS that were *S*. *pneumoniae* PCR-positive but culture-positive for non-*S*. *pneumoniae* specimens (average Ct 40.47, range 39.32–42.33), there was no difference from the Ct values of *S*. *pneumoniae* PCR-positive but culture-negative DBS specimens (average Ct 40.36, range 38.85–45). This contrasts with *S*. *pneumoniae* PCR- and culture-positive DBS specimens where the average Ct was lower at 35.77 (range 33.16–39.31). For two DBS that did meet criteria for a positive result, where *S*. *pneumoniae* was detected in 2/3 replicates with a Ct value of <40, one was from a normal healthy control and one was from a subject who was culture-positive for *H*. *influenzae* type b. Both were negative on repeat testing.

These issues illustrate the difficulties with intra- and inter-user reliability as well as with interpretation of a positive test. Our definition of a positive requiring 2/3 replicates to have Ct counts <40 was based on our validation studies and was potentially too strict, compared to previous studies utilizing molecular methods [26–29], and thereby may have limited the number of specimens deemed positive by molecular detection. Our documented LLD for both types of filter paper corresponded with Ct values <40. As these LLDs fell within the range of bacterial counts in bacteremia [30], we hypothesized that rt-PCR assays described in this study should be able to detect the target pathogen from bacteremic specimens in DBS with typical bacterial loads. However, high Ct values can be more problematic in that they can be the result of nonspecific amplification, can be associated with higher rates of false positives due to poor replicate reproducibility, and can result from the presence of PCR inhibitors or target DNA degradation, or low DNA quantity due to suboptimal transport and storage conditions or antimicrobial treatment prior to specimen collection [31]. In addition, high Ct values have been associated with misclassification of pneumococcal specimens in a pneumococcal surveillance program in South Africa, particularly when the Ct values were ≥35 [32]. As mean Ct results in our study among our *S*. *pneumoniae* PCR- and culture*-*positive specimens extended up to 39.31, we may have missed more culture-positive specimens if we had lowered our Ct cutoff to below 40. Conversely, if we had relaxed our positive criteria to include detection in 1/3 replicates, sensitivity would have only increased minimally from 60% to 66.7%.

While initial studies described the substantially increased sensitivity of molecular diagnostics in detecting bacterial pathogens [29], there is also growing evidence that molecular diagnostics is not without its own pitfalls. A systematic review and meta-analysis of the use of blood PCR in the diagnosis of IPD found that the sensitivity and specificity of molecular testing increased as the reference standard relied more on using any culture, serology or clinical impression of the subject for diagnosis of IPD [33]. Thus, as the reference standard became more uncertain, the better PCR performed [33]. This raises the question of whether molecular diagnostics is sufficiently sensitive or specific to replace the reference standard in clinical practice.

The limitations of this study included the small number of *S*. *pneumoniae*-culture positive specimens in our clinical DBS collection, as well as the availability of either Whatman 903 or FTA at the field site, but rarely both, which restricted our ability to directly compare the detection sensitivity of the two filter papers. The two patients who had blood collected on both Whatman 903 and FTA had pneumococcal DNA successfully detected from both types of filter paper. The DNA extraction methods for Whatman 903 and FTA were disparate, and hence these varied methodologies may explain the differences in sensitivity observed. In addition, the prolonged storage of some of the DBS specimens may have affected the DNA content [34]. While we used a limited number of Gram positive and Gram negative pathogens as negative controls, we did not survey a diverse array of bacteria in our validation of spiked samples and hence cross-reactivity of the *lytA* gene with other bacteria is a possibility.

In conclusion, compared to the gold standard of culture, rt-PCR did identify one culture-negative sample on Whatman FTA DBS from a high-risk subject. Hence, there is promise in this surveillance methodology, which presents a potentially useful epidemiological tool in settings where culture is not available. However, nonspecific amplification for *S*. *pneumoniae* was documented in several clinical samples, including specimens positive for non-*S*. *pneumoniae* on culture. With better delineation of results deemed positive by molecular detection, we could improve our acquisition of disease epidemiology in future studies for resource-limited regions such as Nigeria to monitor pneumococcal disease and the impact of pneumococcal vaccination.

## Supporting Information

S1 Appendix DNA extraction from Whatman FTA paper. (DOCX)

S1 Fig Amplification curves for real-time PCR, at 10-fold dilutions from 5x10^4^ CFU/μL to 0.5 CFU/μL for serotype 5 in whole blood. (DOCX)

S1 Table Primer and probe information for real-time PCR detection. (DOCX)

S2 Table Summary of results of *S*. *pneumoniae* detection in 537 clinical dried blood spot specimens. (DOCX)

## Abbreviations

CSF: cerebrospinal fluid
DBS: dried blood spot
FTA: Flinders Technology Associates
LLD: lower limit of detection
IPD: invasive pneumococcal disease
rt-PCR: real-time polymerase chain reaction
