# Supplementary material for: Molecular Detection of Streptococcus pneumoniae on Dried Blood Spots from Febrile Nigerian Children Compared to Culture
Source: PLoS One. 2016 Mar 23;11(3):e0152253. doi: 10.1371/journal.pone.0152253 (PMC4805257; doi:10.1371/journal.pone.0152253)
Supplement: S1 Appendix — (DOCX) [file pone.0152253.s001.docx]

**S1 Appendix**

1. **DNA extraction from Whatman FTA paper**

For DNA extraction from Whatman FTA paper, three different methods were assessed.

*TE buffer extraction method:* Three 3 mm punches were incubated at room temperature with TE buffer for 15 minutes, followed by incubation at 97 ˚C at 15 minutes [[1](#_ENREF_1)].

*Methanol extraction method:* Three 3 mm punches were incubated with 125 µl of methanol for 15 minutes at room temperature, and then air-dried for 30 minutes. DNA was eluted with 100 µl of water by incubating at 97 ˚C for 15 minutes [[2](#_ENREF_2)].

*FTA purification reagent extraction method:* Three 3 mm DBS punches were incubated for 5 minutes at room temperature, and the reagent was discarded and the process repeated for a total of three times. These were then washed twice with 200 µL of TE. Next, 100 µL of PCR water was added, and the DBS were mechanically disrupted with a pipette for 30 seconds to allow for elution of nucleic acid, and this was incubated at 97 ˚C for 15 minutes. The supernatant was then used as a DNA template in the PCR.

1. **Results of DNA extraction from Whatman FTA paper**

**DNA extractions.** Whatman FTA DNA extraction using the TE buffer extraction method did not lead to any detection on real-time PCR. Using the methanol extraction method for serotype 1, Ct counts ranged from 28.47-40.93, whereas using the FTA purification reagent extraction method, Ct ranged from 24.48-39.85. Based on delayed Ct counts, amplification curves with lower height and linear versus exponential appearance using the methanol extraction method (data not shown), we proceeded with the FTA purification reagent extraction method for subsequent DNA extraction from Whatman FTA DBS.

DNA concentrations were measured on a NanoDrop ND-1000 Spectophotometer (NanoDrop Technologies, Wilmington, DE). The median concentration in whole blood specimens was 15.1 ng/µl. DNA concentration for Whatman 903 DBS was a median of 1.7 ng/µL and the median for Whatman FTA DBS was 10.0 ng/µL.
