# Supplementary material for: Molecular Detection of Streptococcus pneumoniae on Dried Blood Spots from Febrile Nigerian Children Compared to Culture
Source: PLoS One. 2016 Mar 23;11(3):e0152253. doi: 10.1371/journal.pone.0152253 (PMC4805257; doi:10.1371/journal.pone.0152253)

**S1 Fig. Amplification curves for real-time PCR, at 10-fold dilutions from 5x10^4^ CFU/µL to 0.5 CFU/µL for serotype 5 in whole blood.**


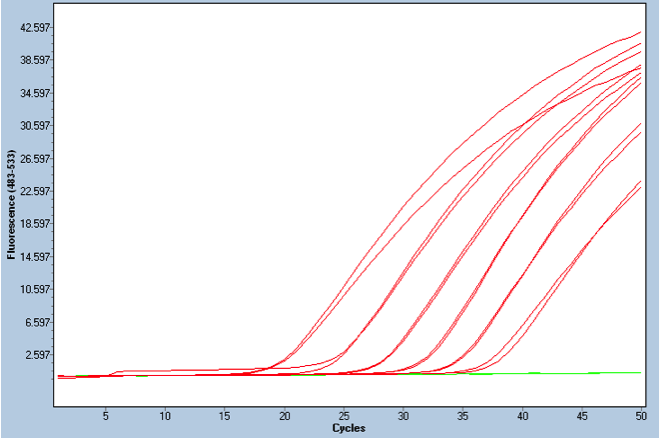

Supplement: S1 Fig — (DOCX) [file pone.0152253.s002.docx]
