# Supplementary material for: Molecular Detection of Streptococcus pneumoniae on Dried Blood Spots from Febrile Nigerian Children Compared to Culture
Source: PLoS One. 2016 Mar 23;11(3):e0152253. doi: 10.1371/journal.pone.0152253 (PMC4805257; doi:10.1371/journal.pone.0152253)
Supplement: S1 Table — (DOCX) [file pone.0152253.s003.docx]

**S1 Table. Primer and probe information for real-time PCR detection**

| Analyte | Primer/ probe | Sequence | Reaction concentration (µM) | Probe modifications |
| --- | --- | --- | --- | --- |
| *S. pneumoniae* (*lytA*) [16, 19] | Forward | ACG CAA TCT AGC AGA TGA AGC A | 0.2 |  |
|  | Reverse | TCG TGC GTT TTA ATT CCA GCT | 0.2 |  |
|  | Probe | TGC CGA AAA CGC ”T” TGA TAC AGG GAG | 0.2 | 5’ FAM, BHQ1 ON “T” |
| Human RNase P [21] | Forward | CCA AGT GTG AGG GCT GAA AAG | 0.32 |  |
|  | Reverse | TGT TGT GGC TGA TGA ACT ATA AAA GG | 0.32 |  |
|  | Probe | CCC CAG TCT CTG TCA GCA CTC CCT TC | 0.08 | 5’ FAM, 3’ BHQ1 |
