# Supplementary material for: Molecular Detection of Streptococcus pneumoniae on Dried Blood Spots from Febrile Nigerian Children Compared to Culture
Source: PLoS One. 2016 Mar 23;11(3):e0152253. doi: 10.1371/journal.pone.0152253 (PMC4805257; doi:10.1371/journal.pone.0152253)
Supplement: S2 Table — (DOCX) [file pone.0152253.s004.docx]

**S2 Table. Summary of results of *S. pneumoniae* detection in 537 clinical dried blood spot specimens**

| Blood culture result | No. of clinical specimens (%) | Detection of *S. pneumoniae* in no. of replicates^a^ | | |
| --- | --- | --- | --- | --- |
|  |  | 1/3 (no. with Ct <40) | 2/3 (no. with both Ct <40) | 3/3 (no. with 2/3 Ct <40) |
| *Streptococcus pneumoniae* | 15 (2.8) | 1 (0)^b^ | - | 9 (9) |
| *Salmonella* Typhi | 15 (2.8) | 2 (2)^c^ | - | - |
| *Salmonella* spp. | 9 (1.7) | - | - | - |
| *Haemophilus* spp. | 8 (1.5) | - | - | 1 (1)^d^ |
| *Staphylococcus aureus* | 4 (0.7) | - | 1 (0)^e^ | - |
| *Neisseria meningitidis* | 4 (0.7) | - | - | - |
| *Escherichia coli* | 3 (0.6) | - | - | - |
| *Candida* spp. | 2 (0.4) | - | - | - |
| *Chryseomonas luteola* | 2 (0.4) | - | - | - |
| Miscellaneous^f^ | 5 (0.9) | - | - | - |
| No data | 84 (15.6) | 3 (1) | - | - |
| Contaminants^g^ | 55 (10.2) | 2 (0)^h^ | 1 (0)^i^ | - |
| Healthy controls | 46 (8.6) | - | - | 1 (1)^j^ |
| No growth | 331 (61.6) | 12 (8) | - | 1 (1)^k^ |
| Total | 537 (100) | 20 (11) | 2 (0) | 12 (12) |

^a^Positive result for *S. pneumoniae* detection was defined as *lytA* gene detected by rt-PCR with Ct value <40 in 2/3 replicates.

^b^Clinical specimen had Ct value of 40.05 in 1/3 replicates. The specimen was negative on repeat rt-PCR testing.

^c^Clinical specimens each had Ct values of 39.32 and 39.85 in 1/3 replicates. One of these specimens was negative on repeat rt-PCR testing, the other was not re-tested.

^d^Clinical specimen was positive for *Haemophilus influenzae* type b on culture. The specimen was negative on repeat rt-PCR testing.

^e^Clinical specimen had Ct values in 2/3 replicates of 39.69 and 40.34, respectively. The specimen was negative on repeat rt-PCR testing.

^f^*Citrobacter* spp. (1), *Enterobacter* spp. (1), *Enterococcus* spp. (1), *Klebsiella* spp. (1), *Pseudomonas* spp. (1).

^g^Contaminants: alpha-hemolytic *Streptococcus* (2), *Bacillus* spp. (13), coagulase-negative *Staphylococcus* (35), diphtheroid (1), *Micrococcus* spp. (2), non-hemolytic *Streptococcus* (1), *Pantoea* spp. (1).

^h^Clinical specimens were alpha-hemolytic *Streptococcus* (Ct value 40.74 in 1/3 replicates) and coagulase-negative *Staphylococcus* (Ct value 42.33 in 1/3 replicates). Both specimens were negative on repeat rt-PCR testing.

^i^Clinical specimen was coagulase-negative *Staphylococcus* (Ct values in 2/3 replicates of 38.34 and 40.21, respectively). The specimen was negative on repeat rt-PCR testing.

^j^Subject was a healthy neonatal control. The specimen was negative on repeat rt-PCR testing.

^k^Subject was a high-risk febrile patient. The specimen was positive (Ct <40 in 3/3 replicates) on repeat rt-PCR testing.
